# New views on the complex interplay between degeneration and autoimmunity in multiple sclerosis

**DOI:** 10.3389/fncel.2024.1426231

**Published:** 2024-08-05

**Authors:** Peter K. Stys, Shigeki Tsutsui, Arie R. Gafson, Bert A. ‘t Hart, Shibeshih Belachew, Jeroen J. G. Geurts

**Affiliations:** ^1^Department of Clinical Neurosciences, Hotchkiss Brain Institute, Cumming School of Medicine, University of Calgary, Calgary, AB, Canada; ^2^Biogen Digital Health, Biogen, Cambridge, MA, United States; ^3^Department of Anatomy and Neurosciences, Amsterdam University Medical Centers (location VUmc), Amsterdam, Netherlands; ^4^TheraPanacea, Paris, France; ^5^Indivi (DBA of Healios AG), Basel, Switzerland

**Keywords:** myelin, B cell, protein misfolding, prion, Epstein–Barr virus

## Abstract

Multiple sclerosis (MS) is a frequently disabling neurological disorder characterized by symptoms, clinical signs and imaging abnormalities that typically fluctuate over time, affecting any level of the CNS. Prominent lymphocytic inflammation, many genetic susceptibility variants involving immune pathways, as well as potent responses of the neuroinflammatory component to immunomodulating drugs, have led to the natural conclusion that this disease is driven by a primary autoimmune process. In this Hypothesis and Theory article, we discuss emerging data that cast doubt on this assumption. After three decades of therapeutic experience, what has become clear is that potent immune modulators are highly effective at suppressing inflammatory relapses, yet exhibit very limited effects on the later progressive phase of MS. Moreover, neuropathological examination of MS tissue indicates that degeneration, CNS atrophy, and myelin loss are most prominent in the progressive stage, when lymphocytic inflammation paradoxically wanes. Finally, emerging clinical observations such as “progression independent of relapse activity” and “silent progression,” now thought to take hold very early in the course, together argue that an underlying “cytodegenerative” process, likely targeting the myelinating unit, may in fact represent the most proximal step in a complex pathophysiological cascade exacerbated by an autoimmune inflammatory overlay. Parallels are drawn with more traditional neurodegenerative disorders, where a progressive proteopathy with prion-like propagation of toxic misfolded species is now known to play a key role. A potentially pivotal contribution of the Epstein–Barr virus and B cells in this process is also discussed.

## Introduction

Multiple sclerosis (MS) was identified as a clinical entity almost two centuries ago and to this day remains somewhat of an enigma in terms of its fundamental cause (Clanet, 2008; Gafson et al., 2012). MS afflicts ≈2.8 million people worldwide (Walton et al., 2020) and is one of the commonest causes of neurological disability among young adults with important geographical differences (Compston and Coles, 2008; GBD 2016 Multiple Sclerosis Collaborators, 2019). MS is somewhat unusual among neurological disorders as it may present with a relapsing–remitting oscillatory course. The pathological hallmarks of relapsing MS are prominent inflammatory foci mainly evident in white matter, with lesions or “plaques” of demyelination characteristically disseminated in space and time. In a minority of patients, MS begins with a progressive course from onset where inflammation may be less prominent, though still plays a role in disability progression (Hughes et al., 2018). Importantly, with time the majority of relapse-onset patients will transition to a clinically progressive course [known as secondary progressive MS (SPMS)] characterized by cytodegeneration, while the inflammatory component wanes with age (Compston and Coles, 2008).

The dichotomic subtyping of MS into “relapsing” and “progressive” forms is somewhat arbitrary, as it is now clear that degeneration and inflammation contribute to disability and pathology at all stages (Hughes et al., 2018; Kappos et al., 2020; Lublin et al., 2022). It is highly likely that the various phases of MS thus represent a continuum of the same disease, where autoimmune inflammation is most prominent early, gradually wanes with age, thus unmasking a progressive underlay of chronic cytodegenerative processes that are present from disease inception; recent consensus appears to be moving in this direction (Kuhlmann et al., 2023).

A decade or so ago, data began to emerge raising questions about the generally-accepted autoimmune etiology of MS, discussed in an opinion paper where we argued that MS might instead be a primary degenerative disorder (Stys et al., 2012). The intervening years have seen accumulating evidence in support of this alternate view of the disease. The aim of this commentary is to provide an update on this more recent evidence and to propose how our understanding of MS pathophysiology should evolve. As the reader will note, many of the headings are stated as questions themselves, emphasizing how much more still needs to be learned. Nevertheless, we argue that a “cytodegeneration” of the myelinating unit may be the most proximal event, which could be potentially driven by a protein misfolding mechanism like most other neurodegenerative disorders. And like such disorders, we will discuss recent evidence describing how progression of pathology throughout the CNS could be based on prion-like spread of proteopathic seeds. The apparently essential role of Epstein–Barr virus is also summarized, including potential mechanisms for how this virus could trigger such a cytodegenerative proteopathy.

## Mechanisms of myelin injury

Multiple sclerosis lesions are characteristically perivascular and exhibit prominent demyelination and bystander axonal damage. The prominent lymphocytic inflammation—at least in the earlier relapsing phase of the disease—is traditionally thought to be the primary insult with immune cells recruited into the CNS to induce recurrent chronic injury (Box 1). Although traditionally considered an inflammatory disorder of white matter, more recently it has become apparent that gray matter damage is also prominent and widespread, though the immune responses are less pronounced in this region, and therefore the radiological and pathological signatures more subtle; virtually all gray matter areas can be involved including cerebral cortex, hippocampus, deep gray matter nuclei, cerebellum and even spinal gray matter (Calabrese et al., 2015; Möck et al., 2021; Ontaneda et al., 2021). Notably, while neuronal elements (axons, neurons, dendrites, and synapses) are also damaged to a significant extent (Albert et al., 2017; Möck et al., 2021), demyelination is a constant finding in both white and gray matter regions pointing to the myelinating unit (the myelin sheath and oligodendrocyte) as a key target in this disease.

While the cause of MS is still unknown even after decades of intensive investigation, the prominence of lymphocytic inflammation, particularly in the earlier relapsing–remitting inflammatory stage, naturally implicates adaptive autoimmunity as a primary driving force. Together with the observation that CNS myelin appears to be a major target, this spurred development of various animal models, with the experimental autoimmune encephalomyelitis (EAE) mouse model being the most popular (Baxter, 2007). EAE is predicated on a primary anti-myelin T cell response and therefore disease is most commonly induced by inoculating mice with myelin peptides together with adjuvant to stimulate an immune reaction against these extrinsically-supplied myelin antigens (Robinson et al., 2014). The ensuing pathology, largely consisting of macrophage and lymphocyte infiltration of mainly the spinal cord, recapitulates the inflammatory pathology of acute MS lesions in a number of respects, but differs from MS in important ways: many EAE variants exhibit only limited demyelination confined to the spinal cord white matter, contrasting with extensive brain pathology also prominently affecting cortical gray matter in MS. Moreover, EAE is characterized by a CD4 T lymphocytic preponderance in contrast to CD8 T cells in MS lesions (Day, 2005). Conceptually however, EAE presupposes that MS is also driven by a primary autoimmune attack originating in the periphery, and if this assumption proves incorrect, then this model may be fundamentally misleading with respect to the true etiology of MS.

Recently, a hybrid model (“cuprizone autoimmune encephalitis” or CAE) combining the myelin-toxic effects of cuprizone (Zirngibl et al., 2022) together with an immune stimulation identical to EAE but without extrinsic myelin peptides, provided proof-of-principle for the notion that a primary biochemical “dysmyelination” can result in a very typical inflammatory demyelinating white matter lesion in an immune-stimulated host (Caprariello et al., 2018). Although the pathology in CAE was mainly targeted to the corpus callosum where cuprizone-mediated white matter injury is focused, very prominent lymphocytic inflammation, demyelination and axonal injury were observed that closely resembled an active MS lesion. In contrast to EAE where the primary stimulus is immune upregulation in the presence of extrinsic myelin antigens, the CAE model illustrates that MS-like pathology can also be generated by an upstream intrinsic myelin injury, releasing antigens which secondarily trigger white matter inflammation in an immune-predisposed host. This is an important insight because it sets the stage, at least in principle, for MS potentially being triggered by subtle injury to CNS myelin rather than by a primary autoimmune assault.

**BOX 1 The immunobiology of MS**

The immune-mediated biology of MS identifies a prominent role of peripherally-derived T and B cells in acute active demyelinating lesions, with microglia, macrophages and astrocytes mainly responsible for chronic inflammation (Rodríguez Murúa et al., 2022; Aliyu et al., 2024). Conventional thinking posits that a primary defect in adaptive immunity promotes recruitment and entry of peripherally activated lymphocytes into the CNS across a disrupted blood–brain barrier where a complex cascade of autoimmune/inflammatory processes culminates in edema, and destruction of myelin, axons and oligodendrocytes (for detailed reviews see Dendrou et al., 2015; Rodríguez Murúa et al., 2022). Early in the course, remyelination of surviving axons can be robust, underpinning clinical remission. However, recurrent bouts of such inflammation and the toxic milieu that ensues, are thought to produce cumulative damage to myelin, oligodendrocytes and neurons, leading to progressive degeneration, atrophy and increasing disability, exacerbated by failure of myelin repair. Modern immunosuppressive medications are highly effective at curbing bouts of inflammation, but have surprisingly limited effectiveness on disease progression. To account for the continuing degeneration and atrophy in later stages, in the face of waning adaptive inflammation as the disease progresses, it has been proposed that the inflammation becomes “trapped” behind the blood–brain barrier, continuing to damage the CNS, but now being inaccessible to peripherally-administered immunosuppressants. Together with still prominent lymphocytic infiltration and inflammation in progressive MS, being particularly pronounced in the meninges and perivascular spaces, leads some investigators to conclude that inflammation primarily drives demyelination and degeneration at all stages of this disease (Lassmann, 2018).

## Disease-modifying treatments

The success of the EAE model in recapitulating many autoimmune inflammatory aspects of relapsing MS spurred the development of many anti-inflammatory drugs. Thus, the mainstay of treatment for MS today is disease-modifying immunomodulation chiefly targeting various aspects of T and B cell pathobiology (Yong and Yong, 2022), which has proven very effective at suppressing acute inflammatory disease (Hauser et al., 2020). Indeed, in more extreme cases with very aggressive inflammatory activity, a total “reset” of the patient’s immune system by autologous bone marrow transplantation has shown remarkable efficacy at stopping new relapses or MRI lesions and even modestly reversing clinical disability (Rush et al., 2019), though currently this aggressive intervention is recommended only for patients with very active inflammatory disease (Miller et al., 2021).

**Figure 1:**
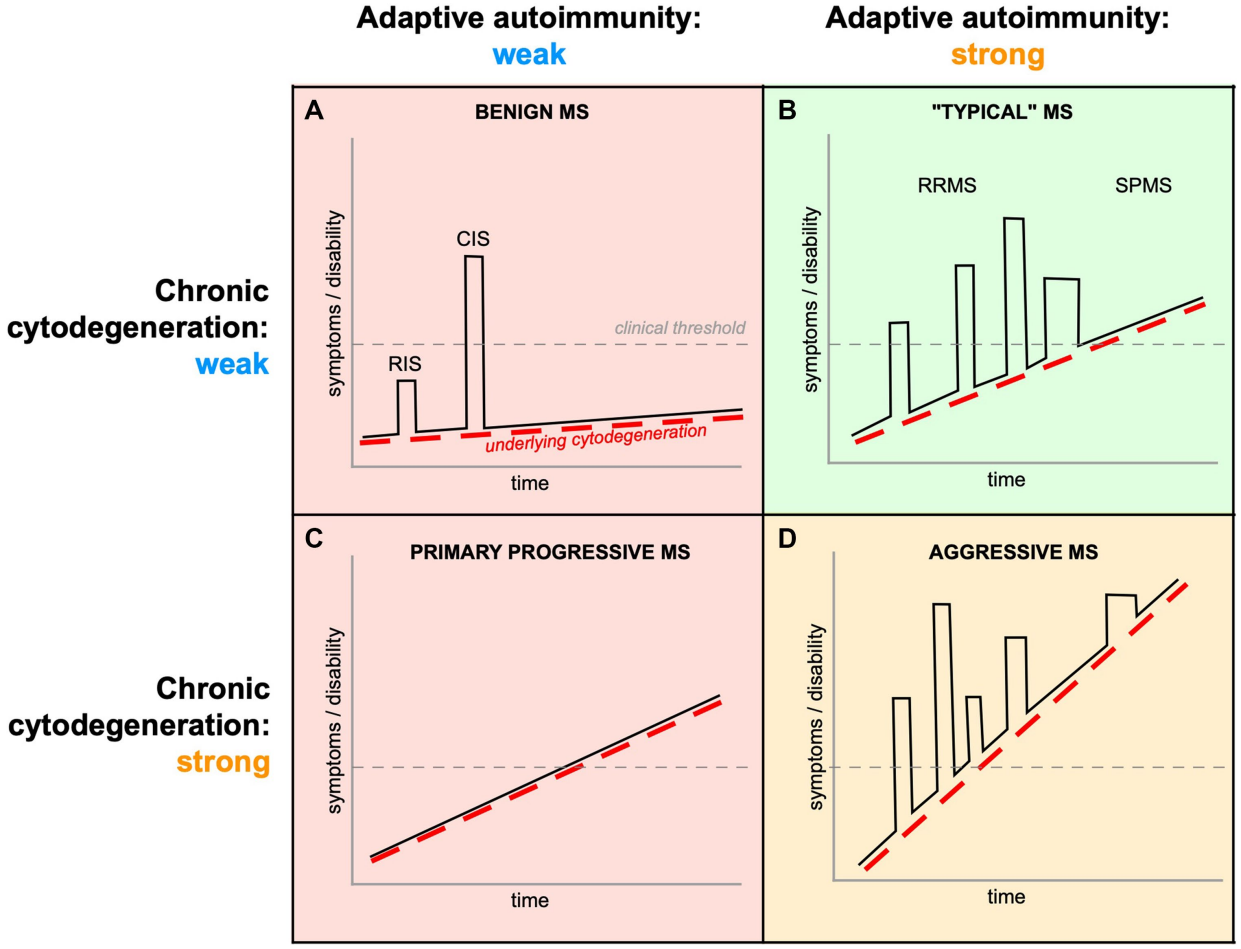
Complex interplay of peripherally-driven acute inflammation and chronic CNS cytodegeneration in MS. All phases of MS exhibit varying intensities of both components, and it is the final “convolution” of the two that determines the clinical phenotype. **(A)** If both components are weak, the mean rate of progression (dashed red line) will be slow, and inflammatory attacks infrequent (CIS) or even subclinical (RIS). **(B)** Even if the underlying cytodegeneration is moderate, more aggressive autoimmunity will result in more frequent inflammatory relapses (RRMS), which may contribute to a more rapid rate of progression because of the additional tissue damage from acute inflammatory episodes. With immune senescence, many relapsing patients transition into a course of disease where only PIRA drives disability worsening (SPMS). **(C)** If the cytodegeneration is strong from onset but with muted autoimmunity, the disease assumes a monotonic progressive trajectory (PPMS). **(D)** Aggressive MS (Iacobaeus et al., 2020) could result from a double-hit of vigorous adaptive autoimmunity on a background of strong cytodegeneration, with both processes contributing by additive mechanisms. Therefore, both acute inflammatory relapses (“relapse associated worsening,” RAW) and underlying primary cytodegeneration (PIRA) contribute to CNS tissue damage and disability (Portaccio et al., 2024), making it very difficult to disentangle their respective contributions. Importantly, current therapies mainly address mechanisms related to acute/adaptive autoimmunity (green shading), and are expected to be largely ineffective (red) or only partially effective (orange) when immune dysregulation is relatively less important.

Despite disease-modifying therapies being highly effective at arresting acute and peripherally-driven inflammation, benefits on progressive mechanisms of the disease—those most responsible for irreversible disability accumulation (Bjartmar et al., 2003)—appear to be very limited (Filippini et al., 2017; Chataway et al., 2024). Intriguingly, emerging evidence suggests that some newer agents such as ocrelizumab (a B cell-depleting monoclonal antibody) and siponimod (a sphingosine 1-phosphate receptor modulator with anti-inflammatory and neuroprotective properties) are beneficial in progressive MS, potentially with effects independent of suppressing acute inflammation (Montalban et al., 2017; Kappos et al., 2018; Arnold et al., 2022). For most disease-modifying drugs, however, the adage “no inflammation, little benefit” remains largely true (Lublin et al., 2022). Notably, a slowing or even reversal of disability stemming from potent medical immunosuppression or bone marrow transplantation should not be necessarily taken as evidence of a *primary* immune pathogenesis, because reducing a toxic inflammatory overlay could independently slow a picture of underlying progressive degeneration. Taken together, if a primary autoimmune hypothesis of MS pathogenesis is correct, it is surprising that potent anti-inflammation would not be more beneficial for the progressive phase. This paradox arises from the inherent complexity of MS pathogenesis which we argue is driven by a “convolution” of relapsing/acute adaptive autoimmunity and chronic cytodegeneration (cyto-is used here rather than neuro-to underscore the point that myelin/oligodendrocytes are the likely primary targets of this process), including an elaborate innate immune response (Figure 1).

## “Inside-Out” vs. “Outside-In”

**Figure 2:**
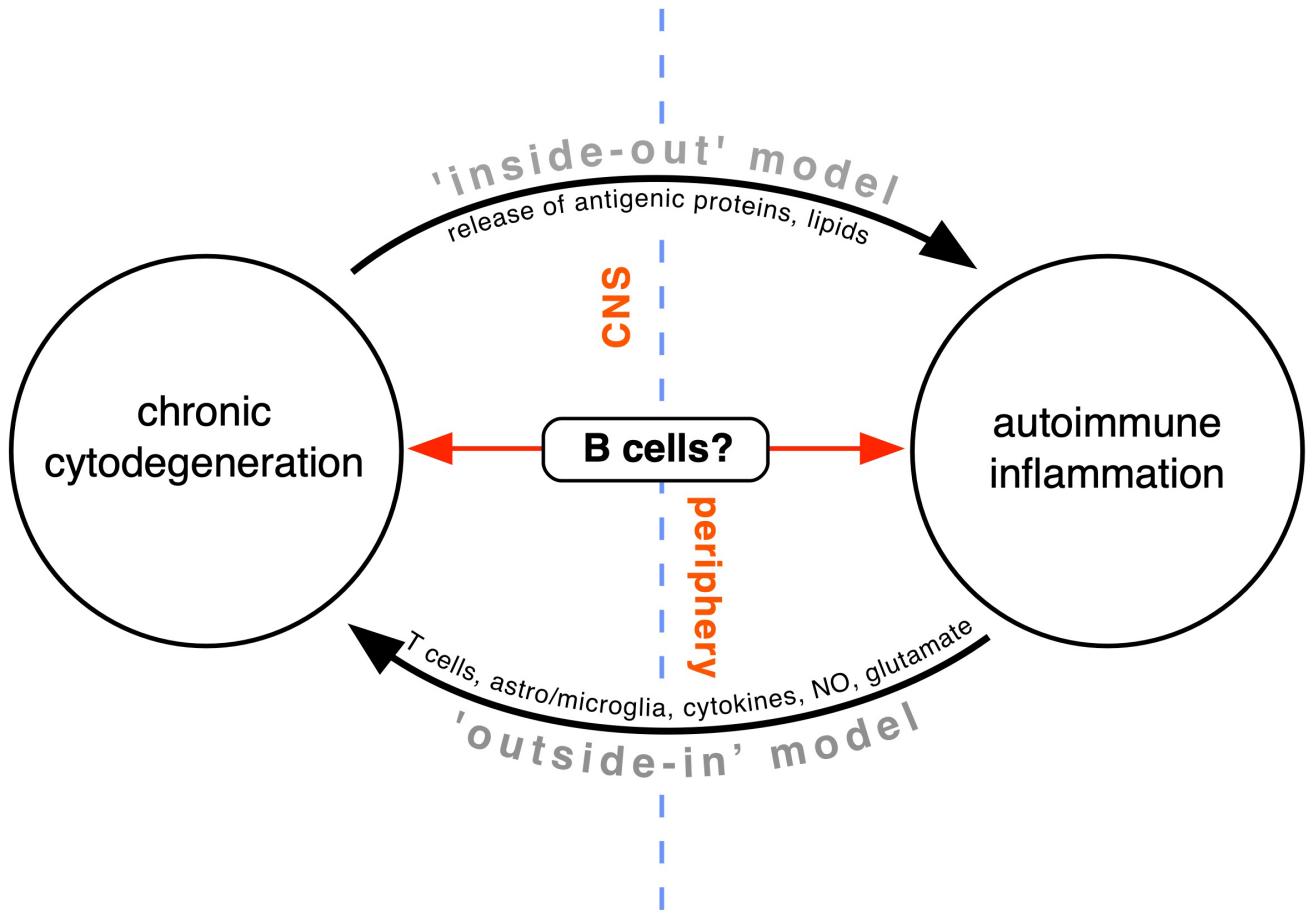
Competing theories of MS pathogenesis. Conventional teaching holds that a peripheral T and B cell-dependent immune defect directed against the CNS white matter is primarily responsible (“outside-in model”). In our opinion, this model fails to adequately explain the progressive phase of MS. Instead, the “inside-out” model proposes that MS is a primary degeneration of the myelinating unit of unknown cause, with antigenic debris triggering an important but secondary autoimmune response in the immune-dysregulated host. Recent evidence suggests that B cells might lie at the interface of the two processes, on the one hand generating toxins that contribute to tissue cytodegeneration, and on the other, conspiring with other immune elements to drive autoimmune inflammation (modified from Stys et al., 2012).

Because CNS pathology, especially that of white matter, so often involves damage to both myelin, glia, and axons, universally accompanied by an immune response, it is extremely difficult—but at the same time very important—to disentangle the precise sequence of events that trigger MS. The challenge is well illustrated by contrasting EAE and CAE summarized above. Even though the location of major pathology differs (mainly spinal cord in EAE vs. corpus callosum in CAE), in the former, a deliberately orchestrated primary autoimmune process directed at myelin antigens results in lymphocytic infiltration, secondary injury to white matter and a universal innate response, whereas in CAE, where the primary insult is a biochemical one deliberately targeting intrinsic CNS myelin, following immune stimulation, the resulting lesion is pathologically similar (Day, 2005; Caprariello et al., 2018). Without *a priori* knowledge of what insults were applied, and in which order, simply observing the final histological lesion renders it impossible to infer the underlying sequence of events nor the initial trigger. This is the situation that researchers face when trying to understand the fundamental pathomechanisms of MS, with the challenge summarized in Figure 2.

**Figure 3:**
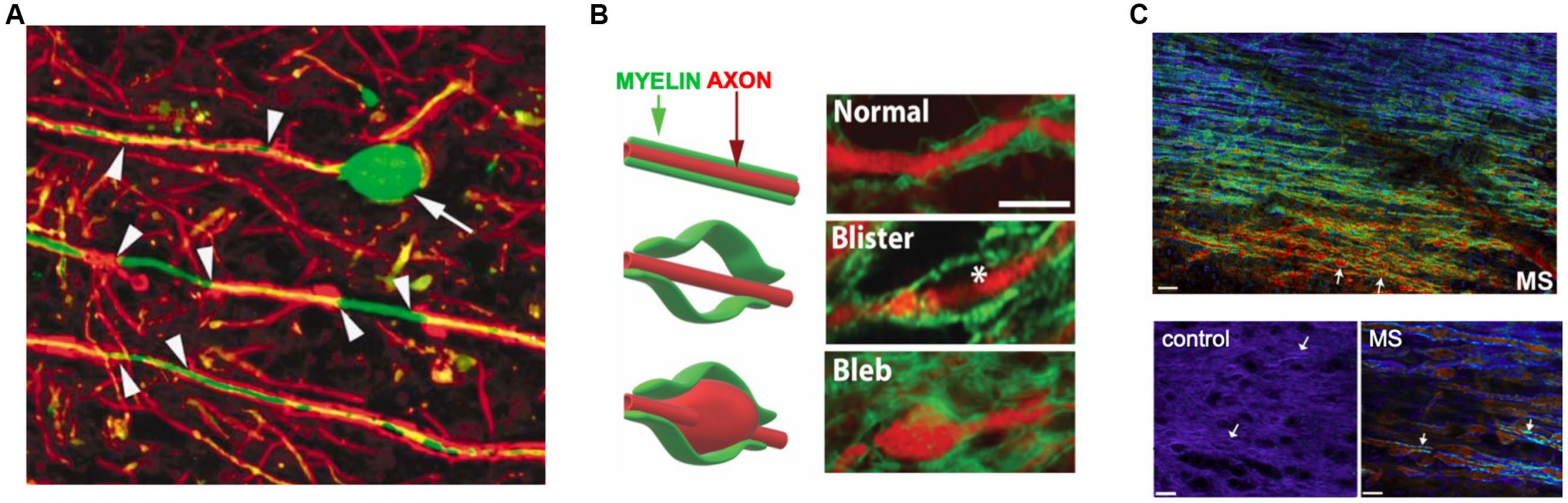
Subtle axo-myelinic pathology in non-lesional MS white matter. **(A)** Segmental demyelination of internodes (arrowheads) and swelling/transection of axons (arrow) are characteristically seen at the periphery of MS plaques, but also in normal-appearing white matter (NAWM) well away from demyelinating inflammatory lesions. **(B)** NAWM also exhibits axo-myelinic injury, with some fibers showing expansion of myelin only (“blisters”), others swelling of both axon and overlying myelin (“blebs”). **(C)** Spectral micrographs of periventricular white matter show a gradient of myelin pathology extending from the ventricle (warmer colors indicate more abnormal but still-intact myelin). Higher power views show normal homogeneous myelin from control vs. highly heterogeneous myelin sheaths (arrows) in axons exhibiting swelling as in panels **(A,B)**. Taken together these data indicate subtle but widespread pathology of axons and myelin in regions with little evidence of lymphocytic inflammation. Modified with permission from Trapp et al. (1998), Luchicchi et al. (2021), and Teo et al. (2021), respectively.

A fundamentally important debate in the field revolves around the “outside-in” (whereby a primary defect in the peripheral immune system promotes invasion of the CNS by autoreactive immune cells) vs. “inside-out” (arguing that a primary CNS degeneration triggers secondary autoimmune inflammation in an immune-predisposed patient) hypothesis (Sen et al., 2020; ‘t Hart et al., 2021; Giovannoni et al., 2022). This debate is reflected in a number of animal models and several human diseases, including careful analysis of human MS (Figure 3; Prineas, 1975; Barnett and Prineas, 2004; Henderson et al., 2009). Regarding the former, transgenic mice that exhibit delayed demyelination and white matter degeneration from a variety of causes (e.g., myelin gene defects, peroxisome-deficient or α-synuclein overexpressing oligodendrocytes, or disruption of their gap junctions, to name a few) frequently show a secondary T- and occasionally a concomitant B-lymphocytic inflammation (Ip et al., 2006; Kassmann et al., 2007; Wieser et al., 2013; Wasseff and Scherer, 2015; Groh et al., 2016; Traka et al., 2016; Williams et al., 2020). Interestingly, direct induction of oligodendrocyte cell death, with resultant demyelination, may not trigger an autoreactive T- or B-cell response (Locatelli et al., 2012; Gritsch et al., 2014). Together these observations suggest that not just any degeneration of myelin, but a specific modification of its constituents is required to elicit a secondary adaptive immune response.

Citrullination of arginine residues in myelin basic protein (MBP)—and likely other CNS proteins as well—seems to be one important post-translational modification associated with inflammatory demyelination. In the CAE model, pharmacological inhibition of citrullination is a potent suppressor of inflammatory demyelination after an immune stimulation, in the absence of any direct anti-inflammatory effect by the inhibitors themselves, indicating that proteins modified in this manner can be potent antigenic targets (Caprariello et al., 2018). Moreover, there is a direct correlation between the degree of MBP citrullination and the aggressiveness of white matter inflammatory activity in MS patients (Moscarello et al., 2007). Importantly however, animal studies collectively emphasize two key principles: first, an adaptive T- and B-cell response can be elicited by myelin that degenerates in a specific way, without any deliberate upregulation of the systemic immune system. Second, that such a reactive lymphocytic infiltration can exacerbate degeneration of white matter elements.

A number of diseases can exhibit similarities to MS, both relapsing–remitting or primary progressive, often to the point of misdiagnosis even by specialists (Table 1). Of note, many of these cases exhibit CSF oligoclonal bands, Gd-enhancing lesions on MRI and clinical response to immunosuppression—all features very typical of MS—indicating that secondary immune responses to a primary trigger can play a significant role in the final clinical phenotype. Importantly, as in the animal studies described above, it is highly likely that this immune response is secondary to a known underlying genetic/biochemical defect that results in degeneration of white matter. One interpretation that is often cited regarding such case reports is that these were examples of a dysmyelination resulting from a defined gene defect or an acquired/sporadic condition, with coincidental MS (Pfeffer et al., 2013; Cloake et al., 2018; Bargiela and Chinnery, 2019). Because the cause of MS is unknown, and because we do not have a specific test to exclude this disease with certainty, this conclusion can never be completely dismissed. However in our opinion, the low probability of MS coincidence with another rare disease, together with the animal studies where MS is never a consideration, makes it more likely that these human examples align with an “inside-out” model (Figure 2), where a primary biochemical derangement of myelin, coupled with an immune predisposition, culminated in a MS-like phenotype.

**Table 1:** Examples of human diseases where a known abnormality (often genetic, but can be acquired) results in white matter pathology and secondary inflammation which can mimic MS.

Disease	Defect	Selected references
Pelizaeus–Merzbacher disease, spastic paraplegia type 2, other PLP mutations	Proteolipid protein 1 gene mutations	Cloake et al. (2018); Gorman et al. (2007); Warshawsky et al. (2005); Qendro et al. (2017); Rubegni et al. (2017)
Harding’s syndrome	Inherited mitochondrial disorder	Bargiela and Chinnery (2019); Beckmann et al. (2021); Joshi and Kermode (2019); Kovacs et al. (2005); Palace (2009)
X-linked adrenoleukodystrophy	ABCD1 gene mutations resulting in abnormalities of very long chain fatty acid metabolism	Di Filippo et al. (2011); Dooley and Wright (1985); Stockler et al. (1993)
Krabbe’s globoid cell leukodystrophy	Galactocerebrosidase (GALC) gene mutations	Tomás et al. (2015)
Hereditary diffuse leukoencephalopathy with spheroids	CSF1R gene mutations	Sundal et al. (2015)
X-linked Charcot–Marie–Tooth disease	Connexin-32 gap junction (GJB1) gene mutation	Isoardo et al. (2005)
Progressive external ophthalmoplegia	Inherited mitochondrial disorder	Gaetani et al. (2016); Patel et al. (2019)
Gerstmann–Sträussler–Scheinker syndrome	Prion protein (PRNP) gene mutation	Karmon et al. (2011)
Vitamin B12 deficiency	Low serum B12, cause unknown	Reynolds et al. (1991); Reynolds (1992)
Miscellaneous	Various	Natowicz and Bejjani (1994); Romagnolo et al. (2014); Weisfeld-Adams et al. (2015); Laurencin et al. (2016)

To further support the above argument, consider the adrenoleukodystrophy/adrenomyeloneuropathy disease spectrum. Neurologically, ALD/AMN can manifest with highly variable phenotypes, ranging from very aggressive and rapidly fatal inflammatory CNS demyelination in young boys to a more slowly progressive adult form either with cerebral white matter inflammatory lesions, or a more indolent progressive myelopathy and peripheral neuropathy (Moser, 1997). Examples exist of brothers, harboring the same mutation, with one afflicted by the aggressive childhood form while the other suffering from a slowly progressive adrenomyeloneuropathy (Palakuzhiyil et al., 2020). Indeed, there are case reports of monozygotic twins harboring the exact same mutation, both with elevated serum very long chain fatty acids (indicating a similar metabolic defect of peroxisomal lipid processing), where one boy is affected clinically and radiologically, while his twin brother is completely normal (Korenke et al., 1996). Such clinical discordance has also been reported for the adult adrenomyeloneuropathic variant (Sobue et al., 1994). Why there should be such striking differences in genetically identical individuals, in a disease caused by the same mutation, is unknown, but likely involves important epigenetic and environmental influences. Also of interest is the observation that mouse models of ALD do not develop cerebral inflammation, but most resemble the non-inflammatory myeloneuropathic phenotype (Pujol et al., 2002). An argument can therefore be made that the “real” ALD is actually the adrenomyeloneuropathic form, with the more aggressive cerebral ALD of young boys driven by a superimposed autoimmune inflammation that is secondary to the myelin lipid derangement. Indeed, it has been reasonably suggested that abnormal lipids, which are known to be antigenic (Kanter et al., 2006), might act as a trigger for an autoimmune response in the human ALD brain (Moser et al., 2007). Similar familial discordance in other inherited disorders further reinforces this notion. For instance, two siblings both harboring the same mitochondrial mutation causing Leber’s hereditary optic neuropathy had markedly different clinical courses: the brother exhibited typical progressive optic neuropathy whereas the sister suffered from a relapsing–remitting MS-like illness (Joshi and Kermode, 2019). One could argue that the higher predisposition to autoimmune disease in women (Cooper and Stroehla, 2003) prompted the sister to react to the subtle white matter damage induced by this mutation in an inflammatory MS-like manner, whereas the brother proceeded along a degenerative path in the absence of the autoimmune overlay. Thus, the ultimate clinical phenotype in a disease where both white matter cytodegeneration and adaptive autoimmune inflammation conspire in the pathogenesis is determined by the vigor of either or both processes, i.e., by a “convolution” of the two. We point out the potential parallels between ALD/AMN and inflammatory relapsing–remitting/primary progressive MS, also at times manifesting as highly aggressive tumefactive disease (Marburg variant). This notion sets the stage for our theoretical dissection of the complex pathogenesis of MS in the next section.

## Is MS a primary cytodegeneration?

In the previous section, we outlined the principle in animal models and humans, of how a primary biochemical disturbance of white matter can trigger a potentially important secondary autoimmune inflammatory response, and indeed, how these two phenomena almost universally co-exist. Whether such an “inside-out” mechanism applies to MS is currently unknown, but here we will discuss recent data that we believe lend increasing support to this notion. While there is now little doubt that a degenerative process operates not only in the later progressive phase of the disease, but also likely from the earliest stages of relapsing inflammatory presentations, what is fiercely debated is whether a primary autoimmune inflammation also drives the continuing CNS cytodegeneration and CNS atrophy (Sen et al., 2020; ‘t Hart et al., 2021; Giovannoni et al., 2022). The limited efficacy of even potent immunosuppression in the later stages (Wolinsky et al., 2020) is likely due to a waning of the acute/adaptive auto-immune component, rather than restricted access of biologicals to the CNS (as an example of adequate access of biologicals to the brain, systemic administration of anti-amyloid antibodies in Alzheimer’s has now been shown to be effective at reducing amyloid load (Jucker and Walker, 2023; Pang et al., 2024), and this in brains that arguably harbor less inflammation and have a tighter blood–brain barrier than in MS). Moreover, bypassing the blood–brain barrier altogether by direct intrathecal administration of the B cell-depleting agent rituximab was also ineffective in progressive MS (Komori et al., 2016; Bonnan et al., 2021) further arguing against inadequate CNS penetration and trapped inflammation as the primary driver of progression. However, one factor responsible for the limited effectiveness of B cell depletors in progressive MS may be resistance of leptomeningeal inflammation to even intrathecally administered agents for reasons that are not understood (Bhargava et al., 2019).

In the more common relapsing–remitting form of MS, if an ongoing acute/adaptive immune response were the primary driver of later progression, atrophy and disability, it would stand to reason that there should be a strong correlation between inflammatory relapses and disability throughout the disease course. The advent of potent anti-inflammatory agents with their strong effects on reducing annualized relapsed rates has advanced the concept of “progression independent of relapse activity” (PIRA) (Lublin et al., 2022). Indeed, recent large studies have now confirmed that a substantial portion (50% or more) of disability accrued even in relapsing MS occurs due to PIRA, i.e., *not* due to incomplete recovery from an inflammatory attack (Graf et al., 2021; Lublin et al., 2022; Portaccio et al., 2024). Remarkably PIRA-like disability accumulation was also observed in pediatric MS raising the possibility that an underlying degenerative process can start very early (De Stefano et al., 2010; Disanto et al., 2016; Dahlke et al., 2021), and is consistent with the idea of a primary cytodegeneration as the initiator of the disease. Another group independently also concluded that disability progression and brain atrophy occur earlier than in the classical secondary progressive stage, with many relapsing patients exhibiting insidious progression without clinical or radiographic evidence of inflammatory disease activity, coining the term “silent progression” (Cree et al., 2019). What underpins progression at the tissue, cellular and molecular levels is still unknown, but taken together, emerging data strongly point to an underlying chronic cytodegenerative process starting early and operating largely independently of overt inflammation.

One compelling histopathological correlate of disability progression is the “slowly-expanding lesion,” characterized by a presumably “inactive” gliotic demyelinated center surrounded by a rim of activated microglia and macrophages containing degenerated myelin, injured axons and often iron deposition (Dal-Bianco et al., 2017; Lassmann, 2018; Jäckle et al., 2020; Pukoli and Vécsei, 2023). The latter is important as it allows the serial imaging of these lesions *in vivo* by MRI, revealing that many slowly expand over time (Dal-Bianco et al., 2017; Elliott et al., 2019; Zheng et al., 2020). These chronic active MS lesions may also harbor at their core (but not at their expanding edges) a perivascular cuff of CNS tissue-resident B and T cells (Machado-Santos et al., 2018; Fransen et al., 2020). Notably absent from areas of active demyelination are significant numbers of lymphocytes, and any lymphocytic infiltrates that were present were distant from regions of myelin and axonal injury (Machado-Santos et al., 2018). The robust presence of innate immune elements at the leading edges led some to conclude that in these chronic active MS lesions, there is a switch with the innate rather than the adaptive immune system taking over to propagate the pathology (Weiner, 2008). This is possible, but given that innate inflammation in the form of microglial activation and macrophage infiltration is a universal response to most types of CNS injury (Rivest, 2009), we would argue that it remains equally plausible that in MS microglia/macrophages are instead reacting to some persistent underlying pathological process in an attempt to limit and repair the expanding white matter damage. Support for this notion comes from instructive pathological feature found at the other end of the temporal spectrum, the so-called “pre-active” lesion, consisting of small clusters of activated microglia seen throughout the MS NAWM, accompanied by axonal and oligodendroglial injury (van Noort et al., 2011; Kuhlmann et al., 2017). In the very early stages, microglial nodules are seen without lymphocytic infiltration (Singh et al., 2013) suggesting that these innate immune cells were reacting to a subtle early pathology that predated the arrival of T or B cells. It is important to note that microglial nodules are not unique to MS, and are also seen in brain trauma, stroke and even normal aging, where they are thought to play a reactive role to minor myelin injury (Kleinberger et al., 2017; Lewcock et al., 2020). Together these observations are consistent with an important concept: microglia are the earliest responders to a primary multifocal myelin injury in the MS white matter, and importantly, this injury/response seems to precede lymphocytic infiltration, arguing against adaptive immune cells being the primary drivers of white matter injury.

If an underlying primary pathology is responsible, what type of early process could be operating in the area of myelin degeneration that does not exhibit signatures of an autoimmune lymphocytic-driven mechanism, or any detectable infectious or other obvious etiology? A re-analysis of a longitudinal MRI dataset on several hundred MS patients (Elliott et al., 2021) confirmed and extended previous reports (Filippi et al., 1998; Pike et al., 2000; Fazekas et al., 2002) indicating that areas of NAWM, where a typical MS lesion will form in the future, exhibited significant abnormalities on T2 and magnetization transfer up to 2 years prior. Such “precursory MS lesions” imply chronic focal pathology that develops well in advance of a full-blown inflammatory MS plaque. What was unexpected was the finding of “mirror” precursory lesions with similar abnormalities to the ipsilateral pre-lesion, but occurring instead in a spatially-matched location in the *contralateral* hemisphere. In contrast to an earlier study that also found such matched contralateral lesions (Werring et al., 2000), this more recent report detected such mirror precursory lesions well before a T2 lesion formed in the index hemisphere, precluding the previous explanation of axonal transection and Wallerian degeneration as a possible contributor to the contralateral mirror abnormality. While the ipsilateral precursory lesion could be explained by an early focal invasion by immune cells that remain dormant for years, the contralateral mirror lesions are harder to ascribe to such a mechanism. What pathological process could be responsible for a very slow, multifocal white matter degeneration that seems to track along connected pathways? The answer is not known at this time, but other classic neurodegenerations could offer a clue.

## Could MS be a proteopathy?

Misfolded protein pathology has now been conclusively demonstrated in all major neurodegenerative diseases involving normally-expressed CNS proteins such as Aβ, α-synuclein, tau, TDP-43, huntingtin, and PrP^c^. Interestingly, under specific laboratory conditions—typically involving transgenic animals expressing the species-matched target protein—pathology characteristic of the originating disease can be transmitted to recipient hosts or even cultured cells (Jucker and Walker, 2018; Scheckel and Aguzzi, 2018; Carlson and Prusiner, 2021). This has also occurred in humans, such as iatrogenic Creutzfeld-Jakob disease (Brown et al., 2012), variant CJD in the context of bovine spongiform encephalopathy (Diack et al., 2014), Lewy body pathology spreading to transplanted fetal neurons (Li et al., 2008) and possibly even Alzheimer’s or cerebral amyloid pathology from injection with contaminated human growth hormone extracts (Purro et al., 2018; Banerjee et al., 2024) or neurosurgical procedures (Panteleienko et al., 2024), respectively. Such transmission, first elucidated for the prion protein in a classic series of experiments (Gajdusek et al., 1966; Gibbs et al., 1968; Telling et al., 1994), is now generally considered to be based on a prion-like propagation of misfolded proteins from affected to naïve host, be it a cell, laboratory animal or human. Once “infected,” either by exogenously supplied transmissible seed (e.g., scrapie inoculation by oral or intraperitoneal administration; Prusiner et al., 1985; Aucouturier et al., 2001; Prinz et al., 2003) or a stochastic conversion of an intrinsic CNS protein, protein misfolding pathology is thought to propagate via interconnected pathways (Jucker and Walker, 2018; Jaunmuktane and Brandner, 2020). In the case of animals inoculated peripherally with scrapie, the agent propagates along peripheral sensory and autonomic nerves in the peritoneum to enter the spinal cord and medulla, and then ascends to invade the brain (Kimberlin and Walker, 1982). Similar to scrapie, α-synuclein pathology spreads in the peripheral and enteric nervous systems, then to CNS, whereas Alzheimer’s tau pathology propagates within the CNS only, beginning in the brainstem then involving cortical areas in a characteristic pattern (Braak and Del Tredici, 2016; De La-Rocque et al., 2021).

If MS were also a degenerative proteopathy, such trans-callosal prion-like spread could readily explain the precursory mirror lesions described above. In addition to multifocal lesions in MS, in a recent study using fluorescence spectroscopy using the solvatochromic lipid probe Nile Red, we reported diffuse changes in myelin lipid polarity in otherwise normal-appearing MS white matter, which was particularly pronounced in the periventricular areas, again supporting the notion of a myelin toxin circulating in the CSF. This technique estimated that the dielectric constant of myelin is increased by ≈10% in MS brain, which would translate into a global 10% reduction of conduction velocity in white matter axons (Teo et al., 2021). These observations emphasized how axons that have intact-looking myelin sheaths could exhibit abnormal conduction properties owing to changes in myelin polarity. Such subtle but widespread alterations of conduction velocity, which would perturb network synchronization in the brain, could be the substrate of frequent and disabling non-focal MS symptoms such as fatigue, mood disorders and cognitive decline (Kalb et al., 2019; Manjaly et al., 2019; Benedict et al., 2020; Capone et al., 2020; McGinley et al., 2021; Raimo et al., 2021).

We have preliminary evidence that transmission of pathology recapitulating abnormalities found in non-lesional MS NAWM occurs in humanized transgenic mice after intracerebral inoculation with MS brain homogenates (Tsutsui et al., 2019). The responsible agent is not known, but we have very recent evidence for presence of amyloid deposition and misfolded proteolipid protein in the brains of progressive MS patients supporting the proteopathic theory (Tsutsui et al., 2024). Finally, it is interesting to speculate that early and extensive thalamic atrophy in MS (Amin and Ontaneda, 2020; Ontaneda et al., 2021) could be due to its rich connectivity, making it a prime target for prion-like agents converging from many brain regions. However, the overall patterns of MS pathology hint at additional routes of propagation discussed below.

If the notion of MS being a primary proteopathy is correct, this raises interesting and unexpected new therapeutic directions modeled on what is being developed for other neurodegenerative diseases such as Alzheimer’s. Recent (albeit modest) successes with amyloid-clearing immunotherapies suggest that reducing the load of potentially pathogenic oligomers or higher-order aggregates may be beneficial, and at the same time provides proof-of-principle that these species are at least partially responsible for promoting the degenerative process (Jucker and Walker, 2023). While we are still a long way away from pinpointing an analogous pathogenic species in MS, once identified, a specific immunotherapy targeting the relevant misfolded proteins, rather than downstream immune effectors as is the current approach, could be more effective in the progressive stages of MS.

## Speculation on the role of B cells

Unlike other neurodegenerative processes, in general, the distribution of MS pathology is more widespread, affecting all regions of the CNS. One consistent pattern of pathology has emerged: there is a strong association of demyelination with proximity to CSF-containing spaces, including the subpial cortex, CSF-filled perivascular Virchow-Robin spaces (part of the brain’s glymphatic system; Eide and Ringstad, 2024), peri-ventricular regions (lateral, third and fourth ventricles) and even the central canal of the spinal cord (Pardini et al., 2021). This “surface-in” pattern strongly suggests that a toxin circulates in the CSF of MS patients to induce myelin damage in the adjacent parenchyma. Neither the nature nor the source of this toxin are known, but could hold the key to the pathophysiology of this disease. One clue is the frequent spatial association between areas of myelin injury and B cell accumulation. A number of studies report frequent collections of B cells (diffuse distribution, more focal aggregates or even “follicle-like” structures) in up to 40% of MS patients at various stages of disease (Jain and Yong, 2021). This leptomeningeal B cell inflammation can be seen around the brain and spinal cord. What is notable is the very frequent spatial correlation with these B cell collections of myelin injury in the subpial cortex as well as spinal cord white matter, raising the possibility that these cells release a myelinotoxic substance (Magliozzi et al., 2007; Howell et al., 2011; Reali et al., 2020; Jain and Yong, 2021). This factor appears to be specific to MS because even more prominent meningeal inflammation in the context of bacterial meningitis for instance, does not induce such pathology (Junker et al., 2020). Release of immune effectors and/or inflammatory polarization of microglia is a logical suspicion, but there may be a more intriguing mechanism at play as suggested by an interesting series of papers by Lisak et al. (2012, 2017). These investigators reported that circulating B cells from MS patients maintained in culture secrete a factor that is toxic to rodent and human neurons and oligodendrocytes. This factor is not complement, cytokine or immunoglobulin, and appears to be packaged into exosomes (Benjamins et al., 2019); currently its nature is unknown.

Taken together, the above observations suggest that B cells might play a key upstream role in MS pathogenesis, and quite unexpectedly, could be stimulated to upregulate machinery unrelated to their traditional immune functions (Lisak et al., 2012, 2017). This is further supported by observations that the frequent oligoclonal immunoglobulins produced behind the blood brain barrier in MS may be non-specific, targeting general cellular debris (Brändle et al., 2016; Hohlfeld et al., 2016; Winger and Zamvil, 2016). Indeed, atacicept, designed to suppress antibody production, was ineffective in a RRMS clinical trial (Kappos et al., 2014). Intriguingly however, very recent data on anti-CD20 B cell depleting agents might be showing some efficacy in progressive MS, although the effect of peripheral B cell depletion on PIRA cannot be interpreted as independent of its effect on silencing acute/adaptive inflammatory activity (Kappos et al., 2020). To what extent systemic infusion of anti-CD20 monoclonal antibodies may deplete B cells or CD20-expressing T cells (Sabatino et al., 2019; von Essen et al., 2019; Ochs et al., 2022) within the CNS parenchyma remains to be established. One could speculate that a key treatment strategy for progressive MS could involve targeting CNS-resident B cells, not aiming to suppress their traditional immunological (mis)behavior, but instead to quell a yet-to-be-discovered unconventional action, possibly related to secretion of an MS-specific myelin toxin into the CSF. It is for these reasons that we positioned B cells at the interface between the CNS and the periphery in Figure 2, as these cells, or more likely, specifically modified subpopulations thereof, could act as a key bridge between acute/adaptive auto-immune attacks and a progressive cytodegenerative process within the CNS.

If indeed, as emerging evidence is suggesting, modified B cells occupy a cardinal upstream position in the complex disease cascade, what might influence them to assume such pathological behavior? As with most biological systems, genetics and environment combine to produce a final phenotype, and MS is no exception. Concordance among monozygotic twins is ≈25% (in fact lower than in migraine, schizophrenia and even infectious diseases such as poliomyelitis and tuberculosis, see below; Ramagopalan et al., 2008), which also implies that identical twins are *discordant* for MS in 75% of cases. This means that genetics certainly plays a role in disease causation, but it is not sufficient, nor is it even the major influence. To date, large studies by the International Multiple Sclerosis Genetics Consortium have identified over 200 independent genome-wide variants associated with MS susceptibility, which mostly involve immune system pathways (International MSGC, 2019; Goris et al., 2022). Given that MS exhibits prominent autoimmune inflammation, this should come as no surprise. Of all the genetic variations identified to date, MS stands alone as a degenerative CNS disorder with no mutation that “causes” MS; this is in stark contrast to Alzheimer’s, Parkinson’s, ALS, tauopathies and prionopathies, all of which have well-defined mutations that cause familial forms of these diseases. We would therefore argue that genetic associations have shed light on the *susceptibility* of developing MS, but have not illuminated the fundamental cause of the disease. As an example, APOE ε4 homozygosity confers a > 90% lifetime risk of developing Alzheimer’s (Liu et al., 2013)—MS has no genetic variation with such a potent effect on disease risk—yet no one argues that APOE ε4 causes Alzheimer’s, only that it strongly modulates the complex processes leading to dementia. One could take this argument further and draw parallels with diseases unequivocally known to be caused by an exogenous agent, such as tuberculosis. Host responses to exogenous pathogens are highly variable (other prominent examples include malaria, hepatitis C and indeed SARS-Cov-2 being the most poignant recent example). In the case of TB, there is a very strong genetic basis of susceptibility to this bacterium, with resistance developing among the indigenous north American population after European colonization, owing to the high initial mortality and therefore rapid selection against susceptibility genes (Abel et al., 2018). Interestingly, just like with MS, the concordance of TB is higher in monozygotic than in dizygotic twins (Kallman and Reisner, 1943). Could MS be “caused” by an exogenous agent, with the widely variable clinical phenotypes determined by host responses and susceptibility, in turn programmed by genetics and lifestyle factors? Adding to the complexity, an exogenous agent may be causative (like the TB bacillus) or simply additive, exacerbating an inflammatory response to some other unrelated underlying insult (Ma et al., 2023). Finally, not all genetic roads lead to the immune system, and the heterogeneity of MS outcome also points toward intrinsic CNS molecular targets (International Multiple Sclerosis Genetics Consortium, 2021; Jokubaitis et al., 2023).

## MS and the Epstein–Barr virus

A number of infectious agents (mainly viruses) have been proposed as etiological factors of MS. This notion was bolstered by epidemiological studies of MS “outbreaks,” with perhaps the best studied being the example of the Faroe Islands (Kurtzke and Hyllested, 1979, 1986). The local population of these isolated islands is of northern European ancestry and therefore expected to have a relatively high prevalence of MS if genetics played a dominant role. Yet there were no reported cases until the arrival of British troops during the second world war, shortly after which time an “epidemic” of MS broke out, leading the prominent epidemiologist John Kurtzke to conclude that MS might be a transmissible infectious disease (Kurtzke, 1993). This controversial view was not universally accepted, and it is likely that the peculiar geographical and global circumstances of the day conspired to generate a unique population-level experiment. On the other hand, this does not mean that Kurtzke was wrong, only that such an experiment is impossible to replicate. As a result, the search for an infectious viral etiology continued over the years, but a firm causative association has been discounted for most, as no consistent presence of virus, DNA or transcripts has been established in the MS brain that is distinct enough from non-MS controls to provide evidence beyond reasonable doubt (for review see Gilden, 2005; Owens et al., 2011).

The Epstein–Barr virus is one notable exception (Ruprecht, 2020; Bar-Or et al., 2022; Aloisi et al., 2023). EBV infection is generally benign, causing a flu-like illness or mononucleosis usually in childhood or adolescence. Occasionally, it is associated with more serious illnesses such as lymphoma and nasopharyngeal carcinoma, owing to its oncogenic properties and strong tropism for B cells and epithelial cells, respectively (Sathiyamoorthy et al., 2016). The virus is ubiquitous, with 95% of the global population exposed to/infected by EBV (Kuri et al., 2020). Interestingly however, studies have repeatedly shown (Bar-Or et al., 2020, 2022; Houen et al., 2020), including a recently published large comprehensive survey of US military personnel (Bjornevik et al., 2022), that EBV exposure greatly increases the risk of MS, to the point that it might be a *sine qua non* for later MS development. However, ascribing causation to this virus is complicated by the fact that 95% of all humans are seropositive, yet comparatively few develop MS. Therefore, it appears that EBV exposure is necessary but not sufficient. Whether EBV is the cause, or is instead the strongest risk factor, can be debated, but the epidemiology convincingly points to this particular virus as playing a key upstream role in the complex pathophysiological cascade. To shed light on whether EBV infection might be a causative agent after invading the CNS following systemic infection, studies were done looking for evidence of virus in the MS brain. The results are often contradictory, with some investigators reporting abundant EBV infection (Serafini et al., 2007), others reporting presence of EBV in both MS and control brain (Tzartos et al., 2012; Moreno et al., 2018), while others still, reporting negative results (Willis et al., 2009; Peferoen et al., 2010). Using PCR, a very recent study found that EBV is equally detectable in the CSF of MS patients and controls (Lehikoinen et al., 2024). These discrepancies may be partially due to technical factors, but so far, evidence for excess EBV in the MS brain is not convincing.

Much has been made of molecular mimicry—that is, the molecular similarity between a viral component for instance and an endogenous CNS protein leading to mistargeted autoimmunity—in the pathogenesis of MS (Lang et al., 2002; Geginat et al., 2017; Lanz et al., 2022). While this concept is undoubtedly real, what influence it actually exerts on disease is unclear. As we pointed out above, to date no pathogenic antibodies, ones that primarily drive the disease process, have been convincingly detected in MS. We would like to propose instead that molecular mimicry may pose a different type of threat to the CNS: that of prion-like templating. Put another way, if the tertiary structures of two molecules (or supramolecular aggregates) resemble each other closely enough to fool an antibody into cross-reacting, then by the same token such a similarity could prompt an endogenous brain protein into being cross-seeded and templated by an exogenous prion-like element. Templating and conformational change is most efficient between like proteins of the same species, but such a protein and species barrier is certainly not absolute, and cross-seeding between different types of proteins is now well established (Chaudhuri et al., 2019; Ren et al., 2019; Daskalov et al., 2021; Ivanova et al., 2021). Can this concept be extended to proteins from different species? Prion protein conversion in variant CJD after ingestion of tainted beef is the most notable example (Baldwin and Correll, 2019). More interesting is the less dramatic but possibly more relevant finding of cross-seeding and induction of Aβ amyloids by herpes simplex viral glycoproteins, possibly resulting from an innate immune response against viral infection by Aβ’s broad-spectrum antimicrobial properties (Eimer et al., 2018; Itzhaki, 2018; Itzhaki et al., 2020; Wainberg et al., 2021). Varicella-zoster virus (also a herpes virus) has also been shown to induce aggregation of Aβ (Bubak et al., 2020). Indeed, sequence analysis has shown that many viral (mainly glyco-) proteins are amyloidogenic, and may aggregate like Aβ (Tetz and Tetz, 2018), while facilitating intercellular spread of misfolded protein pathology (Liu et al., 2021). Perhaps it is not a coincidence that EBV, the main virus implicated in MS, is also a herpes virus, whose genes code for more than 12 distinct glycoproteins, five of which are essential for infecting B cells (Hutt-Fletcher, 2015). Taken together, is it therefore possible that, like herpes simplex in AD, EBV stimulates production of an amyloidogenic glycoprotein that aggregates and in turn cross-seeds an endogenous brain protein, setting in motion a progressive MS proteopathy? Moreover, could certain strains of EBV induce a unique latency program in B cells (Kang and Kieff, 2015; Kempkes and Robertson, 2015) that is MS-specific, reprogramming these to chronically release toxic amyloidogenic material? Notably, brain-resident astrocytes and microglia might also be susceptible to EBV infection, which would greatly broaden the potential source of deleterious viral products and downstream effects, including reactivation of retroviral elements such as HERV-W (Hassani et al., 2018). The above notion could reconcile the paradox of EBV exposure being a strict requirement for subsequent development of MS, and on the other hand the recent failure of an EBV-specific T-cell therapy (Giovannoni et al., 2024): if early EBV infection triggers a molecular cascade driving MS pathogenesis that no longer requires replication of virus, anti-viral therapies begun once MS is clinically evident may be doomed to failure.

The above hypothetical scenario could explain many paradoxes, the most puzzling being that on the one hand, prior EBV infection is required for the development of MS, yet on the other, presence of virus in the brain (assayed by conventional methods probing for viral nucleic acids) is not convincingly detected (Lassmann et al., 2011). Based on the “protein-only” model of prion replication (Diaz-Espinoza and Soto, 2010), no genetic material is required for propagation, and therefore would not be detectable. Adding to the complexity, if the viral glycoprotein cross-seeds an endogenous brain protein, years later even the viral protein need not be present, as the run-away proteopathy would now involve only endogenous brain protein (s). We conjecture that once established, such a proteopathy would no longer respond to B cell depletors (assuming these cells were the original source of the amyloidogenic seeds) after the cross-seeding event has taken hold. Such a model would also explain the late progressive phase of MS, that resembles traditional neurodegenerative diseases in many ways, and that is largely resistant to anti-inflammatory therapies. Moreover, such endogenous brain proteins, cross-seeded to induce aggregation into toxic oligomers, could become the pernicious species responsible for growth of slowly expanding lesions in progressive MS, or form tiny islands of deposited seed to which microglia react to form nodules in pre-lesions. Intriguingly, such prion-like toxic aggregates could represent the suspected demyelinating toxin circulating in the CSF described above. If true, such a scenario would pose a formidable challenge to the research community, because distinguishing a physiological form of an otherwise normal endogenous brain protein from a pathologically misfolded/aggregated conformer is not trivial. Such challenges have been worked on by investigators from the Alzheimer’s, Parkinson’s and prionopathy fields for decades, and we suggest that emerging methods for interrogating protein misfolding could be very applicable to research into the foundations of MS as well. Finally, EBV might occupy a unique and very strategic position in the MS pathophysiological cascade owing to its effects on the immune system, with this virus also implicated in other autoimmune diseases such as rheumatoid arthritis and systemic lupus (James and Robertson, 2012; Balandraud and Roudier, 2018). Therefore, if the above speculations on this virus’ ability to promote a proteopathy are correct, together with its immunomodulatory properties, EBV could deliver a one-two punch and lie at the root of both the cytodegenerative and dysimmune arms characteristic of MS.

## Future directions

Unraveling the mechanisms of disease pathogenesis in traditional neurodegenerations has been very difficult. However much has been learned along the way, and these insights can inspire future research directions in the field of MS. Specific concepts that we believe may be particularly noteworthy include: (1) Misfolding of innate CNS proteins, which then leads to their aggregation into potentially toxic oligomers or amyloids, leading to a toxic gain-of-function via numerous mechanisms including membrane disruption (Kandel et al., 2017; Ito et al., 2023) (this may be particularly relevant for the destabilization of myelin), synaptic dysfunction (Tu et al., 2014) (this includes impairment of signaling at the axo-myelinic synapse, crucial for survival of the myelinated axon; Micu et al., 2016, 2018), oxidative stress (Cheignon et al., 2018), mitochondrial impairment (de la Cueva et al., 2022) (well documented in the MS brain; Mahad et al., 2008), excitotoxic mechanisms (Armada-Moreira et al., 2020), and the intimate relationship between misfolded protein aggregates and innate inflammation that can further exacerbate tissue injury (Joshi et al., 2014; Kwon and Koh, 2020). (2) One consequence of #1 is a potential alteration of normal protein–protein interactions, which are key to normal cellular physiology. Detailed study of the cellular interactome in MS can shed light on disease mechanisms and point to new therapeutic directions. Protein–protein interactions are potentially druggable, with a number of modulators already in clinical trials, mainly in the field of oncology (Lu et al., 2020). (3) A key property of pathological protein aggregates is their ability to spread in a prion-like manner. Modeling experiments on work done with scrapie, Alzheimer’s, Parkinson’s disease and others, exploring the propensity of transmitting pathology from human material to cells and animals engineered to be susceptible to such spread, would provide strong evidence for the proteopathic theory of MS. We have preliminary observations in support (Tsutsui et al., 2019), and this could be expanded with a more detailed dissection of the precise transmissible species, target elements (for instance, is the myelinating unit most vulnerable?), and mechanisms of transmission and spread. (4) Finally, exploring mechanisms even more upstream, by asking what triggered the initial protein misfolding process, could get at the core of MS pathogenesis. The proposition that herpes simplex virus may be a proximal event in Alzheimer’s pathogenesis (Itzhaki, 2021), juxtaposed against the overwhelming evidence supporting the role of EBV in MS, inspires another compelling research direction. With a better molecular understanding of such specific mechanisms, adjunctive therapies can be developed for a combination treatment strategy, as is the standard approach for many other diseases.

## Conclusion

Multiple sclerosis is a complex disorder that has puzzled the medical and scientific communities for almost two centuries. In the last decade, significant new data have accumulated lending support for an underlying, early cytodegenerative process of unknown nature that exhibits remarkable similarities to other neurodegenerative disorders. What has been abundantly clear is that inflammation is a key component of MS, also contributing to CNS atrophy and disability, particularly in the early relapsing phase. What has become equally clear however, is that autoimmune inflammation is only one part of the equation, with an underlying cytodegeneration beginning early and continuing relentlessly throughout the course of the disease. This adds to the difficulty in reconciling MS under a single pathophysiological umbrella, but also has implications for therapeutic design: this cytodegenerative component is not expected to respond to current anti-inflammatory disease-modifying drugs targeting acute/adaptive peripheral autoimmunity. We argue that viewing MS through the lens of an early onset, slowly progressive cytodegeneration of white and gray matter elements, convolved with a variable overlay of adaptive autoimmunity, could readily explain the entire disease spectrum, and aligns well with emerging new evidence. The question of what might be the proximal trigger is as difficult to uncover as it has been for other neurodegenerative diseases, but a viral etiology, in particular EBV, deserves further scrutiny. Expanding our view beyond the traditional autoimmune etiology is important if we are to gain a deeper understanding of the fundamental mechanisms of this disease. Whether the theory of an underlying proteopathy is correct or not, we would argue that evidence accumulated over the last decade makes this hypothesis a plausible consideration. Importantly, the MS research community could glean valuable concepts and borrow helpful techniques from the neurodegeneration field. New insights gained from such novel approaches could in turn herald an entirely new direction for development of next-generation adjunctive therapies, particularly for the intrinsically progressive nature present throughout all phases and clinical forms of MS.

## Data availability statement

The original contributions presented in the study are included in the article/supplementary material; further inquiries can be directed to the corresponding author.

## Author contributions

PS: Conceptualization, Writing – original draft, Writing – review & editing. ST: Writing – review & editing. AG: Writing – review & editing. B’tH: Writing – review & editing. SB: Writing – review & editing. JG: Writing – review & editing.

## Glossary

Adrenoleukodystrophy/adrenomyeloneuropathy (ALD/AMN): An X-linked disorder caused by a mutation of the ABCD1 gene coding for a peroxisomal fatty acid transporter. The same gene defect can cause a severe and rapidly fatal childhood leukodystrophy or a more indolent adult-onset myelopathy and neuropathy.
Amyotrophic lateral sclerosis (ALS): A progressive fatal neurodegenerative disorder of unknown cause affecting upper and lower motor neurons in the brain and spinal cord.
Cuprizone autoimmune encephalitis (CAE): A variation of EAE where subdemyelinating doses of cuprizone are administered to mice (without exogenous myelin antigens), followed by an immune stimulation. CAE provides proof-of-principle that an *endogenous* disruption of myelin can trigger MS-like brain lesions in an immune-predisposed host.
Clinically isolated syndrome (CIS): A first attack of neurological disability with accompanying white matter lesion(s) on imaging, that may or may not develop into MS characterized by recurrent lesions distributed in space and time.
Creutzfeld-Jakob disease (CJD): A rare and invariably fatal neurodegenerative disease caused by a misfolding of the cellular prion protein into a propagatable and transmissible species. Variant CJD is acquired by ingestion of meat from cattle afflicted by bovine spongiform encephalopathy, with the BSE prion crossing the species barrier and misfolding native human brain PrP^c^.
Cerebrospinal fluid (CSF): Cerebrospinal fluid that bathes the brain and spinal cord, and is thought to contain circulating factor(s) that contribute to MS pathophysiology.
Epstein–Barr virus (EBV): One of several herpes viruses afflicting humans that is globally ubiquitous, and is considered necessary but not sufficient to trigger MS.
Experimental autoimmune encephalomyelitis (EAE): The most popular rodent model of MS employing exogenously-administered myelin antigens together with immune stimulation that replicates some aspects of autoimmune inflammation found in the human disease.
Normal-appearing white matter (NAWM): Normal-appearing white matter in the MS brain appears intact by traditional histological and radiological readouts, but is known to harbor subtle but significant widespread abnormalities of myelin and axons. A similar subtle gray matter pathology has also been described in the MS brain.
Progression independent of relapse activity (PIRA): Progression independent of relapse activity indicating accumulation of irreversible clinical disability and CNS demyelination in the absence of overt inflammatory relapses.
Primary progressive MS (PPMS): Primary progressive MS characterized by monotonic progression of clinical disability and brain atrophy without inflammatory relapses.
Cellular prion protein (PrP^c^): The normally folded physiological form in contrast to disease-associated conformers such as the scrapie isoform.
Relapse-associated worsening (RAW): Clinical progression in MS patients attributable to recurrent inflammatory attacks targeting various regions of the CNS, and inducing bystander damage to axons, myelin and glia.
Radiologically isolated syndrome (RIS): Incidental appearance on MRI scans that resemble typical MS lesions in a patient with no history of matching neurological disability. Likely equivalent to CIS but affecting a non-eloquent area of the CNS.
Relapsing–remitting MS (RRMS): The commonest form at onset characterized by recurrent bouts of inflammatory demyelination at any level of the CNS, including brain, spinal cord and/or optic nerves.
Secondary progressive MS (SPMS): A later phase of RRMS patients who often transition to a progressive course when recurrent inflammatory attacks subside due to immune senescence.
